# Healthy Homes: Repairs and Maintenance in Remote Northern Territory Housing

**DOI:** 10.3390/ijerph22060836

**Published:** 2025-05-26

**Authors:** Liam Grealy, Jiunn-Yih Su, David Thomas

**Affiliations:** Menzies School of Health Research, Charles Darwin University, Darwin, NT 0810, Australia; jiunn-yih.su2@menzies.edu.au (J.-Y.S.); david.thomas@menzies.edu.au (D.T.)

**Keywords:** remote communities, housing policy, evaluation, indigenous governance

## Abstract

This article examines Healthy Homes, a program intended to initiate a new approach to housing repairs and maintenance in remote communities in the Northern Territory of Australia. It argues that while the evidence for associations between poor housing and poor health outcomes is clear, greater attention should be paid to the implementation of health-focused housing interventions. Healthy Homes was examined through interviews with public servants, Aboriginal community-controlled organisation staff, and householders, alongside participant observation during maintenance projects and Condition Assessment Tool inspections. Routine housing, inspections, and expenditure datasets were also analysed. Across 5498 houses subject to Healthy Homes and over a twenty-month period, only 1315 Condition Assessment Tool inspections were completed, which is the key mechanism for generating preventive maintenance work. Expenditure on repairs and maintenance was stable between the old maintenance model and under Healthy Homes. Most Healthy Homes remote housing maintenance contracts were awarded to Aboriginal business enterprises. This article finds that Healthy Homes did not effectively shift remote property management to prioritise preventive maintenance. Issues with data collection and monitoring, program implementation, and contractual arrangements impeded more consistent and effective attention paid to the condition of housing health hardware. Future investment into the implementation of health-focused remote housing preventive maintenance programs must attend to the details of program design, including the data collection processes and contractual terms for service providers.

## 1. Introduction

The ‘Healthy Homes’ program was one part of the Northern Territory (NT) Government’s ‘Our Community. Our Future. Our Homes’ (OCOFOH) remote housing investment package, jointly funded by the Australian and NT governments under the former National Partnership for Remote Housing Northern Territory (2018–2023). Healthy Homes was framed as a new approach to remote housing maintenance, adopting a cyclical and preventive approach and supporting residents to undertake ‘healthy living practices’ (HLPs) [1]. The not-for-profit company Healthabitat refers to Healthy Living Practices (HLPs) as practices that housing should enable residents to undertake to ensure their good health. These include washing people, washing clothes and bedding, removing wastewater safely, and so on. HLPs depend on ‘health hardware’, which refers to the physical equipment needed to ensure that housing and related environments support good health for householders. For example, in relation to the need for residents to safely store and prepare food, health hardware includes functional shelves and cupboards, a stove, and a refrigerator)

Commencing in 2021, the Healthy Homes program applied to 73 NT remote communities, Alice Springs town camps, and Tennant Creek community living areas, with four distinct components: the delivery of Housing for Health (HFH) projects by the not-for-profit organisation Healthabitat in selected remote communities; new contracts for remote housing maintenance services and tenancy management support services; capacity building for householders focused on housing and hygiene; and monitoring and evaluation [1,2]. The program recognised the important relationship between quality housing, including functional health hardware and householder health outcomes, and followed litigation related to the failure of the NT government to provide necessary repairs and maintenance to housing in the communities of Ltyentye Apurte (Santa Teresa) and Laramba [3].

A housing crisis is also a health crisis, and the association between poor housing and negative health impacts for Aboriginal and Torres Strait Islander householders is well established. Brackertz and Wilkinson describe that in the year 2014–2015, 28 per cent of Aboriginal and Torres Strait Islander people over 15 years of age lived in housing with major structural problems, rising to 36 per cent in remote areas [4]. In their narrative literature review of publications addressing the relationship between infectious diseases and maintenance of Aboriginal and Torres Strait Islander housing, Ali et al. found that ‘Gastrointestinal, skin, ear, eye, and respiratory illnesses were all related in various ways to functional health hardware, removal and treatment of sewage, crowding, presence of pests and vermin, and the growth of mould and mildew’ [5,6,7]. Bailie and Wayte characterise the shortcomings of remote community housing in relation to conceptions of ‘adequacy’ that include ‘quality of basic services, materials, facilities and infrastructure; habitability; affordability; accessibility; legal security of tenure; and location and cultural adequacy’ [8].

Crowding, or ‘overcrowding’ as defined by the Australian Bureau of Statistics is a major contributing factor in housing-related health outcomes, as it can increase the transmission of common infectious diseases and parasites [9,10]. The ABS defines overcrowding according to the Canadian National Occupancy Standard (CNOS) as a household needing three or more additional bedrooms to accommodate its usual residents. In their analysis of common childhood illness in remote NT communities in 2004–2005, Bailie et al. found that ongoing crowding undermined the potential for improved health outcomes in communities with new houses [11]. In their review, Ali et al. found that a lack of maintenance in Aboriginal and Torres Strait Islander community housing was associated with gastrointestinal infections, that crowding was associated with skin-related diseases and viral conditions, and that inadequate food preparation and storage areas were associated with diarrhoea [5]. Such infections can ‘ultimately result in chronic sequelae, such as stunting, blindness, hearing loss, rheumatic heart disease and renal failure’ [5].

There are a range of interventions that governments can pursue to improve housing to support Aboriginal and Torres Strait Islander health outcomes. Ware distinguishes between approaches focused on any of infrastructural improvements, addressing behavioural factors, adjustments to policy environments [12]. NT remote housing and infrastructure programs have typically prioritised both new housing construction and the addition of bedrooms to existing housing, recognising the connections between crowding and poor health outcomes—and because crowding is straightforward to measure in comparison with housing quality. In government housing programs, repair and maintenance are often subordinated to capital works, despite their own impact on sustaining health hardware and in effect reducing crowding in more functional houses. Repair and maintenance are less visible and, following occupancy, faults in houses can be more easily attributed to householders. Under-funded and reactive approaches to housing maintenance have had priority—over preventive attention.

Underinvestment in remote housing repair and maintenance and related environmental health interventions has been recognised by numerous reports and reviews over the past two decades, which have recommended programmatic, consistent, and sustained approaches to housing maintenance [13,14,15]. One of the key recommendations of the Commonwealth Government’s National Partnership Agreement for Remote Indigenous Housing (NPARIH) review—‘an increased emphasis on planned cyclic maintenance’ to maintain existing housing stock [16]—echoed a NPARIH goal from a decade earlier, which was to ‘implement robust and standardized Property and Tenancy Management (PTM) of all remote Indigenous housing’ [17].

Healthy Homes was thus notable for responding to such reports, recognising the connections between repair and maintenance and householder health and the importance of preventive maintenance. This article considers findings from a two-year monitoring and evaluation project. It focuses specifically on the remote housing maintenance services contracts established for all communities subject to the program, the component of Healthy Homes with the most potential to positively impact householder health. In doing so, it highlights the significance of service contracts for the efficacy of housing maintenance and environmental health programs in remote housing generally. The broader evaluation found that few contracted service providers developed meaningful interventions focused on householder engagement and health promotion related to healthy living practices (via tenancy management support services contracts). Similarly, while Housing for Health projects make significant improvements in the communities where they are delivered, the broader evaluation determined that this involved a small number of communities during the evaluation period [18]. This article moves beyond repeating the scientific evidence for improving housing in order to improve health outcomes to instead examine the practical details of how the Healthy Homes program—which was explicitly based on this evidence—was administered and implemented. It argues that an effective and health-conferring property management regime requires preventive maintenance and that consistent preventive maintenance relies on well-designed and administered service contracts. Such contracts establish the opportunities and constraints within which organisations can deliver the programs recommended by health practitioners and funded by governments.

## 2. Materials and Methods

### 2.1. Evaluation Approach and Expert Advisory Group

Menzies School of Health Research was employed by the NT Department of Territory Families, Housing and Communities to monitor and evaluate Healthy Homes. The researchers undertook a process evaluation, monitoring the program and providing feedback from its commencement [19], to ‘measure the activities of the program, program quality and who it is reaching’ [20]. The approach also involved aspects of developmental evaluation, including regular feedback to the program developer and key industry stakeholders, and a flexible methodology that responded to restricted access to field sites and datasets [21,22]. The process evaluation plan adopted asked who, what, when, and where questions about Healthy Homes [23], such that ‘attention [was] paid to features of the target group, the implementers or change agents, the frequency of intervention activities, and features of the information imparted’ [24]. This approach was augmented by the researchers’ consultation with Harfield et al.’s ‘Aboriginal and Torres Strait Islander Quality Appraisal Tool’ to ensure that the approach taken, where possible, pursued appropriate research questions and engagement in line with Aboriginal and Torres Strait Islander values and principles for ethical research [25].

The project was informed by an Expert Advisory Group (EAG), including peak body Aboriginal Housing NT (AHNT), the Northern and Central Land Councils, the Arnhem Land Progress Aboriginal Corporation (ALPA), Tangentyere Council Aboriginal Corporation (TCAC), and Yilli Rreung Housing Aboriginal Corporation (YRHAC). All of ALPA, TCAC, and YRHAC held Healthy Homes contracts for remote housing maintenance services. While the inclusion of such organisations in the EAG could suggest a conflict of interest, their participation was important for advising the research on the procurement process and the terms of those contracts, while also providing broader expertise on town camp and remote community housing management. AHNT and the two major land councils are members of the Joint Steering Committee for Remote Housing Northern Territory (JSC-RHNT), which is responsible for overseeing the delivery of remote housing programs under national housing agreements.

### 2.2. Data Sources and Analysis

This article focuses on simple descriptive statistics based on analysis of routine reporting and expenditure datasets related to Healthy Homes (2022–2023) and housing maintenance in NT remote communities more broadly across the period 2013 to 2022. The researchers received Corporate Business Intelligence System (CBIS) datasets drawn from the NT government’s Tenancy Management System (TMS), including rates of overcrowding by community, basic features of housing stock (e.g., dwelling size) and dwelling standard, previous five-year expenditure per dwelling, and inspections data. Subsequent reports provided by the department were generated from the Asset Systems Nexus Program (ASNEX) and related to expenditure under Healthy Homes property maintenance services contracts and maintenance works in remote communities under the prior model contracts.

The evaluation sought to disaggregate NT government expenditure data by work/trade type, based on invoicing data from contracted service providers. Doing so is useful for projecting trade-specific expenditure in future years and can indicate under-investment in certain trade areas where additional investment has the potential to reduce specific infections or illnesses.

**Figure 1:**
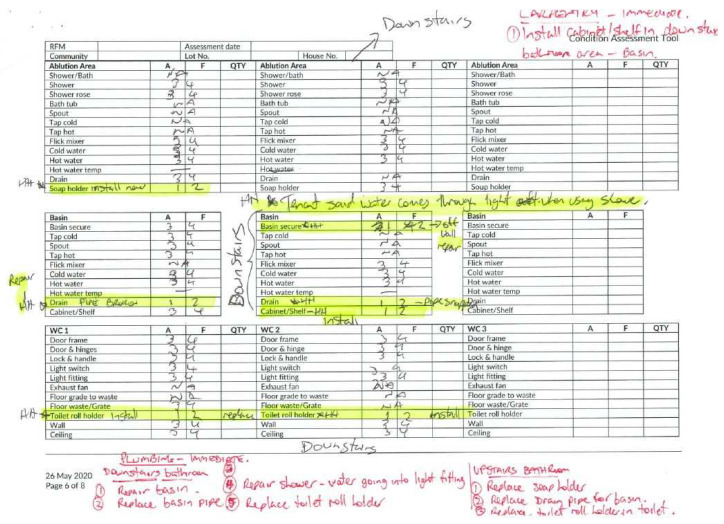
An example of a completed CAT page with work orders.

However, this disaggregation of total repair and maintenance expenditure by work area does not clarify the extent to which Healthy Homes prioritised preventive maintenance over reactive repairs. The key component of Healthy Homes maintenance contracts distinguishing them from the old model was the requirement that contracted service providers undertook annual inspections of all their properties using an NT government ‘Condition Assessment Tool’ (CAT). The CAT is an eight-page paper form used to assess housing function and generate work orders (Figure 1). This cyclical and preventive mechanism aimed to generate maintenance works earlier than would occur by relying on tenants to report faults, thus reducing their severity and improving houses’ functioning overall. Description is provided of the extent to which CAT inspections were undertaken by contracted service providers, which does not itself clarify the functionality of housing health hardware or the proportion of preventive maintenance undertaken across the program.

Documentary analysis of program materials also informed the evaluation, including the interpretation of descriptive statistics, such as Healthy Homes tender documents and contracts between the NT government and service providers under the prior and Healthy Homes regimes. We examined such documents in order to describe key program indicators, including the number and type of contracts awarded, and whether they were awarded to Aboriginal business enterprises; the number of dwellings and the percentage that were overcrowded; the annual expenditure per dwelling and expenditure by work type; and the number of preventive CAT inspections undertaken. The broader evaluation project (not reported here) also conducted formal interviews with NT government public servants, staff of contracted service providers, householders, and other stakeholders including EAG members. This was complemented by participant observation undertaken by the lead researcher in remote communities and town camps while participating in project works.

## 3. Results

### 3.1. Maintenance Contracts

The former remote housing maintenance model, established in 2013–2014, involved a two-tier maintenance approach. Housing Maintenance Officers (HMOs) were employed locally to provide simple repairs that did not require a licensed tradesperson and were complemented by a regional Trade Panel for specialised trade works. Under that model, remote housing maintenance services were divided into tenders for Trade Panel Services and Housing Maintenance Coordination Services. Trade Panel Service contracts were awarded to multiple trade companies within a region of clustered communities.

Healthy Homes reconfigured the contractual model for remote housing repair and maintenance in the NT, with a single contract typically awarded by the NT government for clusters of one to three communities. This approach sought to prioritise the procurement of local Aboriginal business enterprises (ABEs) to deliver maintenance work in specific communities. As of May 2023, 31 contracts for remote housing maintenance services had been awarded to 22 companies, including 25 contracts to 17 ABEs. These contracts covered 49 communities, the Alice Springs town camps, and the Tennant Creek community living areas. Similar results were achieved for remote housing tenancy management support services contracts. Table 1 shows the total housing maintenance services and tenancy management support services contracts awarded under Healthy Homes, whether these were awarded to ABEs or non-Indigenous contractors, and the number of remote communities to which these contracts applied.

In 24 remote communities, Healthy Homes maintenance services contracts were not awarded and the prior trade panel maintenance model continued to operate. In 21 of those 24 communities, the housing maintenance coordinator contract was not renewed.

Under Healthy Homes, while most remote housing maintenance services contracts were awarded to ABEs, interviews with contracted service providers conveyed contractual issues related to total value, contract length, the schedule of rates, approval thresholds for works, and reporting requirements. Following significant program delays, contracts were awarded for short periods—between 15 and 22 months. Some providers reported issues with the total value of contracts, which required costing individual items on a schedule of rates—a list of job types and associated values that sets pricing for invoicing by service providers—to cover preliminaries, overheads, and on-costs, including travel expenses. Incomplete schedules of rates, where unscheduled works could not claim originally agreed-upon cost indexing, meant that ABEs could not always recoup the cost of maintenance work, especially where remoteness challenges to service delivery were greatest. Contracts did not include a guaranteed minimum of work, accounting for inflation was inadequate, and contracts did not include a presumption that the awarded supplier would be preferred for contract renewal. Unless organisations negotiated otherwise, a default contractual requirement to gain approval from the NT government contract superintendent for non-urgent works over AUD 500 created additional administration and delays in the delivery maintenance works.

### 3.2. Expenditure

The evaluation determined that in December 2022 there were 5498 dwellings (houses and units) subject to the Healthy Homes program, including 5084 in remote communities, 298 in Alice Springs town camps, and 116 in Tennant Creek community living areas. Of the total occupied dwellings, 52.9 per cent were overcrowded, ranging from 18.3 per cent of dwellings in Alice Springs town camps to 68.1 per cent in the Arnhem region. Across the five-year period of 2017–2022, the total number of dwellings increased by 421 or 8.3 per cent from the 2017 baseline.

Under Our Community. Our Future. Our Homes, AUD 35 million per annum was budgeted for remote community housing repairs and maintenance. In 2022, the first full calendar year of Healthy Homes, NT government expenditure data indicated that total expenditure under active remote housing maintenance services contracts was AUD 43.12 m. This included AUD 32,238,183 under Healthy Homes contracts and AUD 10,883,019 under ongoing trade panel contracts. From 2015 to 2022 inclusive, the average total expenditure on remote housing maintenance was AUD 40.23 m per annum, indicating consistent expenditure under the initial rollout of Healthy Homes.

**Figure 2:**
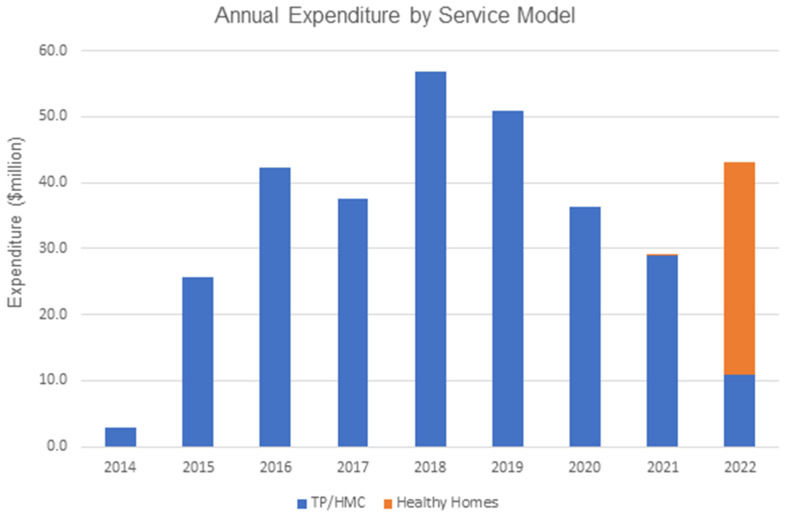
Annual remote housing community maintenance expenditure by service model.

The average per annum expenditure per house across the same period was AUD 7727 (range: AUD 5156 in 2015 to AUD 11,049 in 2018), with an average expenditure per house in the final year (2022) of AUD 7843. Figure 2 shows the annual expenditure by maintenance service model (trade panel and housing maintenance coordinator model and Healthy Homes model) over time from 2014 to 2022. Table 2 compares the average expenditure per house in 2022 under trade panel and Healthy Homes contracts. Healthy Homes contracts applied to a significant majority of houses (4342 of a total 5498) but demonstrated a lower per house annual expenditure.

Analysis of NT government expenditure data alongside historical contracts determined that total expenditure related not only to remote community housing (i.e., public housing for Aboriginal tenants) but also government employee housing in those communities (a total of 1864 in December 2022), a legacy of specified properties in the former trade panel contracts. Analysis of expenditure by individual Healthy Homes contracts indicated that about 10.8 per cent of total expenditure was allocated to the approximately 15.1 per cent of houses in the total stock that were government employee housing (89 of 588 houses across three indicative contracts). Adjusting for this, the average expenditure per house in 2022 was estimated as AUD 6352 on remote public housing and AUD 4399 on government employee housing.

**Figure 3:**
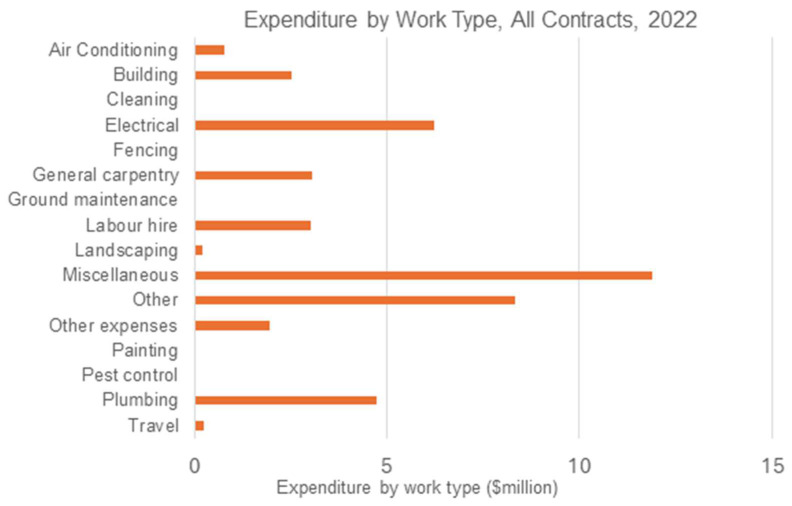
Distribution of total expenditure by work type in 2022.

Figure 3 shows the distribution of expenditure by work type for all active maintenance contracts in 2022. Expenditure on key trades constitutes a significant proportion of total expenditure. Electrical work accounts for AUD 6.2 m; general carpentry accounts for AUD 3 m; and plumbing accounts for AUD 4.7 m of the total AUD 43.1 m.

Also evident in Figure 3 is the significant proportion of work coded to miscellaneous, AUD 11.9 m; other, AUD 8.3 m; and other expenses, AUD 1.9 m. Together, approximately 51.5 per cent of total expenditure in 2022 was coded to one of these categories. Within the expenditure data, ‘other’ includes a number of subcategories: cyclical housing preventative maintenance survey—tradesman; cyclical housing preventive maintenance survey—trainee; unscheduled item—provide quote for scoped works; and non-access to dwelling. Across the period July 2021 to February 2023, a total AUD 11,522,983.74 was categorised as other within the remote housing maintenance data.

### 3.3. Preventive Maintenance

Despite contractual obligations, only a small number of Condition Assessment Tool (CAT) inspections were actually undertaken. Reporting data (CBIS Inspections Completed Report) indicated a total of 143 CAT inspections undertaken from the commencement of Healthy Homes in July 2021 to the end of February 2023. Further investigation of contract expenditure data (using ASNEX Contract Item Usage Detail Reports, coded for work type) across the same period indicated a total 1315 CAT inspections completed across a total 5498 houses included in Healthy Homes. This equated to a CAT inspection being completed in only 23.9 per cent of remote community houses. By February 2023, many of the houses subject to Healthy Homes remote housing maintenance services contracts should have received two CAT inspections, as an annual inspection was required to be undertaken within the first three months of contract commencement.

## 4. Discussion

Across the five-year period of 2017–2022, despite increasing the total number of dwellings by 421 (or 8.3 per cent from the 2017 baseline), the proportion of overcrowded remote community dwellings in the NT only decreased by 2.09 per cent (from 54.99 to 52.90 per cent). This should be compared with the aspiration of Closing the Gap target 9a, which aims to ‘increase the proportion of Aboriginal and Torres Strait Islander people living in appropriately sized (not overcrowded) housing to 88 per cent’ by 2031 [26]. Overcrowding can be reduced by maintaining the condition of health hardware in all houses, such that residents do not congregate in the proportion of houses with working amenities.

### 4.1. Precarious Contracts

The details of maintenance contracts and procurement processes are rarely considered in critical commentaries on public housing neglect or public health analyses of effective interventions for improving householder health outcomes. However, getting such details right—recognising the true cost of remote housing maintenance and the administrative conditions facilitating or impeding service delivery—is centrally important for expanding and sustaining the role played by Aboriginal community-controlled organisations in remote housing management.

Under Healthy Homes, remote housing maintenance services contracts were awarded for between 15 and 22 months. Contracts of this length constitute a precarious contracting regime under which service providers are unable to plan ahead, providing staffing challenges and setting a short period in which to deliver a sustainable and health-conferring program following set-up costs. Contractors reported a range of related issues undermining the sustainable delivery of maintenance works, related to the total value of contracts, schedules of rates, guaranteed minimum work, and administrative burdens regarding departmental approvals for work orders. Moving forward, consultation with contracted service providers is required by the NT government to revise future contacts in ways that enable Aboriginal business enterprises to deliver an effective service in a timely fashion and with due remuneration.

### 4.2. Data Limitations

The goal of Healthy Homes was to prioritise cyclical and preventive repairs and maintenance to improve the condition of houses and to support good health outcomes for householders. While there are thorough expenditure data available, the collection and collation of data on house condition and function and maintenance by trade type are limited. This must be improved to monitor program outputs and investigate any relationship between maintenance, house function, and health outcomes.

NT government datasets do not provide detailed information about the health hardware of dwellings at the time of inspections and their related capacity to support healthy living practices. Expenditure data are only an approximate proxy for the total quantity of housing maintenance services delivered to remote householders, and expenditure is not a good indicator of the quality of maintenance works. Nonetheless, the evaluation analysed expenditure data as both the most detailed representation of the work undertaken through Healthy Homes and due to their interest to the project Expert Advisory Group.

In communities where Healthy Homes remote housing maintenance services contracts were established, maintenance expenditure was similar under the prior model and Healthy Homes. However, it was not possible within NT government datasets to distinguish between responsive repairs and preventive maintenance works generated by undertaking cyclical CAT inspections. Where CAT inspections were undertaken, it was not clear that the information gathered (see the handwritten annotations in Figure 1), as with the survey results of Housing for Health projects, was incorporated into any dataset about the condition or function of remote community housing. The non-completion of CAT inspections compounds the lack of data available to the NT government and community housing providers related to house condition and function and the trade types of work undertaken within the maintenance program. This lack of data undermines the effectiveness of any future asset management strategy by either government or community housing providers. It is also an impediment to any transition of housing to Aboriginal community control according to terms where the maintenance liability has been properly costed.

Routine data is thus inadequate for the NT government to evaluate the impact of the Healthy Homes program (with respect to existing key performance indictors and reporting requirements) and ultimately to determine whether Healthy Homes initiated a new approach to housing repairs and maintenance (and to what extent). At the outset of the monitoring and evaluation project, it was the researchers and the NT government’s shared intention that this would be a two-stage evaluation. Stage 1 was to focus on program implementation, while Stage 2 would proceed to measure the health impacts of the program. Given the partial program implementation (the incomplete award of Healthy Homes contracts across the jurisdiction and the limited application of CAT inspections as the main preventive maintenance mechanism), the researchers determined that it would not be possible to meaningfully evaluate any health impacts of the program.

### 4.3. A Preventive Maintenance Program?

The chief feature that differentiated Healthy Homes as a preventive and cyclical maintenance approach from the prior model was the requirement that contracted service providers use a Condition Assessment Tool (CAT) to conduct inspections to generate preventive maintenance. The data provided to the evaluation offered little evidence that preventive maintenance was undertaken in more than a few locations in the NT.

From July 2021 to February 2023, less than a quarter of houses involved in Healthy Homes were inspected using the CAT. Service providers reported that the NT government inspection tool was confusing and difficult to use, with some providers unaware of the contractual obligation to undertake such inspections. The extent of training provided to contracted service providers regarding the use of the Condition Assessment Tool was also very limited. In order to achieve better outcomes, the program must increase the number of preventive maintenance inspections, revise the inspection tool, improve its integration with wider program maintenance approaches, and provide training to service providers to increase their confidence in applying the CAT tool as required by contract. All of these changes are necessary if Healthy Homes is to become a program that uses inspections proactively to generate preventive maintenance works in order to reduce the negative health impacts of poor housing.

The extent to which Healthy Homes remote housing maintenance services contracts were awarded across NT remote communities is also necessary to acknowledge in relation to any analysis of the past or prospective impact of Healthy Homes on house quality or householder health outcomes. In 24 of 73 remote communities, maintenance services continued to operate according to the old system, less the role played by housing maintenance coordinators. Positive or negative housing outcomes in those contexts do not represent any impact of the new program. Taken together, that is, the partial coverage of Healthy Homes contracts across NT remote communities and the failure of contracted service providers to implement CAT inspections as required, it is reasonable to question any claim that Healthy Homes was, in effect, a preventive maintenance program.

## 5. Conclusions

Healthy Homes did not meet its goal to generalise a preventive maintenance approach across remote housing in the Northern Territory. The program’s intentions—to pursue preventive maintenance focused on maintaining health hardware as delivered by Aboriginal business enterprises—should continue to be pursued. Significant further work is required to align program delivery—from contracts to schedules of rates, inspections processes, data collection and management, inter-departmental collaboration, and sector development—with program aims, such that an ongoing intervention will have positive environmental health impacts on householders. Such contractual and program considerations are relevant beyond remote Indigenous housing in Australia, including for social housing property management in general.

Completed in 2023, the evaluation recommended that the next national housing agreement be established for ten years to allow for longer-term maintenance contracts that would support the development of the Aboriginal community-controlled housing sector, as well as underpinning service continuity for remote householders. In June 2024, the Australian and NT governments published a ten-year Federation Funding Agreement Schedule for remote housing, with AUD 3.98 billion allocated across the period. For the first time, an agreement of this type specified funding to be allocated to property and tenancy management activities (AUD 92 m in 2024–2025) with a specified ‘AUD 10 million each year to develop and implement a program of Cyclical and Preventive Repairs and Maintenance works’ [27]. The evaluation findings are informing the design of the ongoing remote housing maintenance program, including through their consideration by a working group of the Joint Steering Committee for Remote Housing NT.

Our research indicates that to achieve the greatest health gains through a preventive maintenance program, consideration should be given to the details of the terms on which service providers are contracted to deliver repair and maintenance works. This includes designing contracts that support Aboriginal community-controlled organisations to undertake maintenance works effectively and sustainably and in ways that ensure the data collection necessary for all stakeholders to understand the conditions of remote housing. In other words, it is not enough to demonstrate the negative impacts of poor housing on householder health or even to allocate funds for a preventive maintenance program. Governments and associated stakeholders must ensure that the aims of a preventive maintenance program are integrated throughout program contracts and reporting processes, such that those aims might be actioned by contracted service providers and measured by program administrators.

## Figures and Tables

**Table 1:** Healthy Homes contracts awarded as of May 2023.

Contract Type	Aboriginal Business Enterprise (ABE)	Not ABE	Total Awarded	Remote Communities
Housing maintenance services	25	6	31	49
Tenancy management support services	19	6	25	47

**Table 2:** Average expenditure per house by service model in 2022.

	Trade Panel	Healthy Homes	Total
Total expenditure (AUD )	10,883,019	32,238,183	43,121,202
Number of houses	1156	4342	5498
Per house expenditure (AUD )	9414	7425	7843

## Data Availability

The datasets presented in this article are not readily available because they are owned by the NT government. Requests to access the datasets should be directed to the NT government.
